# Engaging the Community: An Interview with Uche Amazigo

**DOI:** 10.1371/journal.pntd.0000268

**Published:** 2008-07-30

**Authors:** Hannah Brown

Walking purposefully towards the shabby grey concrete structure that functions as the main health centre of Kyenjojo district in western Uganda, public health doctor Andrew Byamungu makes a trip he has done many times before since joining the vector control department of the Ugandan Ministry of Health. Fighting his way through the crowds of waiting patients and relatives, he greets the tired-looking health staff who, among their many other duties, are responsible for education and drug distribution in the country's onchocerciasis control programme, which is one of the most advanced of the 19 country projects of the African Programme for Onchocerciasis Control (APOC).

**Image 1:**
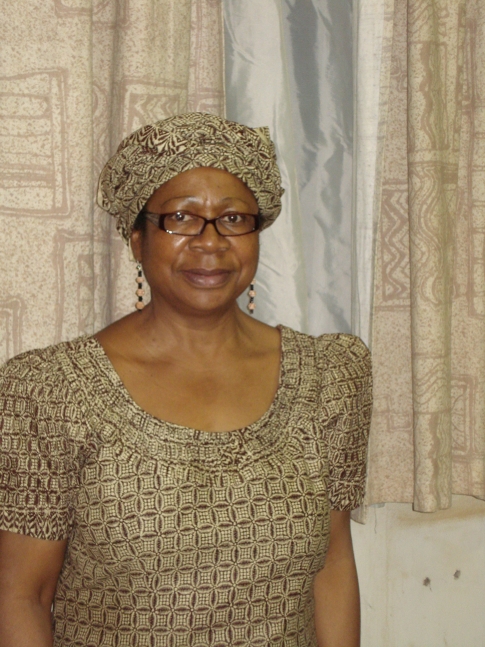
Uche Amazigo

After a brief exchange with the long-serving nurse-cum-midwife, Dr Byamungu explains that the health centre staff want to know if this time he is accompanied by the programme's personable director, Uche Amazigo (Image 1), whose innumerable visits to APOC's project sites—from Uganda's bustling west to remote mountain villages in Cameroon—are just one of the reasons her programme has been lauded as the most successful public health effort in Africa. “Everyone knows Amazigo,” he says with a chuckle.

A soft-spoken, elegant woman in her fifties, Dr Amazigo, who became director of APOC in 2005, developed an interest in onchocerciasis—known colloquially as river blindness because of its most destructive symptom—as she began to learn about the social consequences of the disease from women she met as a result of regular trips into Nigeria's rural southeast. Working as a lecturer on tropical diseases in the university town of Nsukka in the late 1970s, Dr Amazigo had a chance encounter with a pregnant woman who was plagued by the disease's characteristic itchy lesions and striking depigmentation, and whose husband had left her due to these disfiguring effects.

Moved and deeply saddened by the woman's predicament, Dr Amazigo resolved to help pay for her treatment and to learn more about how the disease destroyed lives. She enrolled in a local rural women's group to hear firsthand about the consequences of onchocerciasis, a parasitic infection transmitted through the bite of the blackfly, on important life experiences such as marriage and breast feeding. And, around the same time, she contacted the head of the WHO-associated Special Programme for Research and Training in Tropical Diseases (TDR) and was encouraged to apply for a grant to study the disease properly. She took up the offer, and her research has since helped change international perceptions about the morbidity associated with onchocerciasis. WHO has repeated Dr Amazigo's studies in several other countries, and this data formed the scientific basis for launching APOC in 1995, which Dr Amazigo was invited to join as a scientist in 1996.

APOC's strategy is based on preventive treatment of affected populations with a once-yearly dose of the anti-onchocerciasis drug ivermectin. Thanks to an unlimited donation of the agent by its manufacturer, Merck & Co., APOC's main task has been to work out a way to deliver the treatment to communities in a sustainable and long-term manner. The strategy that has made APOC so successful—one that Dr Amazigo helped develop while working as a scientist at TDR—uses unpaid community workers to supplement the health system by training them to treat themselves and their neighbours and to complete record books to track drug distribution. “All the communities want is for you to come and train their own chosen people. Once you have trained them, you send drugs to the drop-off point, and they will complete drug registers and return them,” Dr Amazigo enthuses. “Communities do the job that the health system should do, but the important thing is that we do it in a way that will make communities feel very proud to be doing it—that they are able to distribute drugs themselves—and proud to serve the community.”

Across 19 sub-Saharan African countries, the remarkable successes achieved by APOC and its less extensive predecessor programme, Onchocerciasis Control Programme (OCP), which focused on 11 countries in West Africa, show that Dr Amazigo's long-term support for this strategy is not misplaced. “You now have countries where onchocerciasis is no longer a public health problem,” she says. “In Burkina Faso, Mali, Senegal, Niger, Guinea, Togo, and Benin it is very hard to find any child going blind from oncho because they have active national surveillance which has maintained the achievements of the OCP.” Some of these countries have also started to reclaim land previously deserted due to the presence of blackfly to grow cotton on a large scale. “Some are up to the level of exporting cotton, so this is a huge economic development,” adds Dr Amazigo.

As APOC's mandate nears its pre-defined endpoint of 2015, Dr Amazigo is now turning her attentions to a new and potentially more ambitious challenge. Using the community-directed treatment strategy APOC has refined, she is now adamant that the programme should become a model for a more general strengthening of health systems in African countries. APOC's experience shows that the system can work, successfully and sustainably, even in the most difficult environments, such as the conflict-scarred and poverty-stricken Democratic Republic of Congo. What is more, says Dr Amazigo, research done during APOC's 10-year operational period shows that community volunteers could actually be more effective than the health system, producing better results and much higher treatment coverage.

For Dr Amazigo, integrating APOC's activities with the health system is her opportunity to leave a lasting legacy, in the shape of improved health services for hundreds of thousands of people. In some countries, notably Dr Byamungu's domain in Uganda, the process has already begun. Community volunteers recruited for APOC are also delivering vitamin A, deworming treatments, and home-based malaria care to their charges since the Ministry of Health adopted APOC's strategy as a model for delivering other simple primary health care interventions in an integrated way. And with the high-level endorsement of WHO Director General Margaret Chan, who told delegates at the 1st Neglected Tropical Diseases Global Meeting of Partners in Geneva in April 2007 that the legacy of onchocerciasis control in Africa was a prime example of the vertical-to-horizontal transition of health programmes that is necessary to make control strategies sustainable in countries with weak health systems, Dr Amazigo is hopeful more will follow.

“Given the lack of human resources, particularly at the peripheral level, how many doctors would like to go and live in a sub-district area? How many nurses are there? Very few. The way to go is to bring the communities to be part of health services,” Dr Amazigo explains. But there is a drawback: community-directed treatment needs patience, time, and commitment. These qualities are often in short supply when it comes to health programme managers focused on short-term results. “Some people want quick results and so they don't have the patience to implement the process,” she laments. “I am not saying it is easy, but I am convinced that this is the way forward.”

